# Positive Parenting Behaviors and Child Development in Ceará, Brazil: A Population-Based Study

**DOI:** 10.3390/children9081246

**Published:** 2022-08-18

**Authors:** Hermano A. L. Rocha, Luciano L. Correia, Álvaro J. M. Leite, Sabrina G. M. O. Rocha, Lucas de S. Albuquerque, Márcia M. T. Machado, Jocileide S. Campos, Anamaria C. e Silva, Christopher R. Sudfeld

**Affiliations:** 1 Department of Global Health and Population, Harvard T. H. Chan School of Public Health, Boston, MA 02115, USA; 2 Department of Maternal and Child Health, Federal University of Ceará, Fortaleza 60020-181, CE, Brazil; 3 Department of Community Health, Federal University of Ceará, Fortaleza 60020-181, CE, Brazil; 4 Integração Saúde Ensino Comunidade, University Center Unichristus, Fortaleza 60020-181, CE, Brazil

**Keywords:** child language, child, preschool, parenting, parent–child relations, Latin America

## Abstract

Parenting practices have been identified as a key determinant of children’s developmental outcomes. The aim of this study was to evaluate the association of parenting practices with child development in a cross-sectional population-based study in a low-income state in northeastern Brazil. The study included data on 3566 caregiver–child pairs, and the children were aged 0–66 months. Positive parenting behaviors (PPBs) were conceptualized in areas of interactive play, social development, and speech and language interactions. Child development was evaluated using the Brazilian Ages and Stages Questionnaire. Linear regression analysis was used to assess the relationships. We found that a greater number of PPBs was associated with better child development domain scores. Among infants < 1 year, each additional PPB was associated with a 0.32 standardized mean difference (SMD) greater communication (95% CI: 0.24–0.41) and 0.38 SMD greater problem-solving scores (95% CI: 0.24–0.52). Among children aged 4–6 years old, each additional PPB was associated with improved communication (SMD: 0.22; 95% CI: 0.13–0.32), problem solving (SMD: 0.21; 95% CI: 0.10–0.32) and personal–social domain scores (SMD: 0.26; 95% CI: 0.17–0.36). Our findings indicate that PPB were robustly associated with better outcomes across developmental domains among Brazilian children. Programs and interventions that support PPB can contribute to improvements in development outcomes.

## 1. Introduction

Globally, it was estimated that 52.9 million children under the age of five were developmentally delayed in 2016, compared to 53.0 in 1990, a very small relative reduction. It was also identified that the vast majority of these children, 95%, live in developing countries [1,2]. In response, the World Health Organization, UNICEF, and the World Bank, in collaboration with the Partnership for Maternal, Newborn and Child Health, the Early Childhood Development Action Network, and partners, developed the Nurturing Care Framework, which includes five components as priorities for supporting children’s development: responsive caregiving, good opportunities for early learning, adequate nutrition, and security and safety [3]. Child development plays an essential role in the course of a person’s life, and studies have found that development in early life is associated with productivity and income generation in adulthood [4,5].

There is a relatively large body of literature, mostly observational studies, that have documented an association between parenting and child health and developmental outcomes [6]. Parenting is usually defined as one of two constructs, either parenting behaviors or parenting beliefs. Parenting behaviors represent a broad construct that reflects the actions taken by parents during the upbringing of their children. Parental beliefs, on the other hand, represent what parents think about themselves, their children, and the process of raising them [7].

Research on positive parental behaviors has demonstrated that greater parental engagement is strongly associated with better child development outcomes, such as a cohort study carried out in England with 5-year-old children [8,9]. Furthermore, speech interactions between parents and their children have been shown to be positively associated with language development, while better play interactions have been associated with improved attachment and motor development [10,11,12]. Nevertheless, the majority of research on parenting practices and children development has been conducted in North America and Europe, and empirical evidence of the association between positive parenting behaviors and motor and cognitive development in Latin America is lacking. A recent systematic review assessing parenting interventions identified that less than 10% of the studies were conducted in Latin America [13]. Moreover, the majority of research assessing parenting behaviors in Latin America has comprised convenience samples or children enrolled in government programs and not population-representative samples [14].

To fill this evidence gap, we performed a cross-sectional population-based survey of children aged 0–6 years residing in the state of Ceará, Brazil. We evaluated the association of positive parenting behaviors (PPBs) with child communication, gross motor, fine motor, problem solving, and personal social development scores. We hypothesized that more PPBs would be associated with better child developmental outcomes.

## 2. Materials and Methods

### 2.1. Study Design

We used data from a cross-sectional study of children who participated in the population-based PESMIC survey (Maternal and Child Health Survey of Ceará, *Pesquisa de Saúde Materno Infantil do Ceará*), realized in Ceará, Brazil [15]. PESMIC is a study on child and maternal health of children up to six years old residing in the state of Ceará. Ceará is in northeastern Brazil and is one of the most impoverished states in the country, with a population of nine million dwellers living in a semiarid climate, with high prevalence of food insecurity. Fortaleza is the urban commercial center and capital city of the state, and there are also rural areas in Ceará, where subsistence farming is the main economic activity [16].

The PESMIC surveys were carried out in the years 1987, 1990, 1994, 2001, 2007, and 2017. Data from the 2017 survey were used for this analysis. The full details of the PESMIC methods can be found elsewhere [15]. One-hundred and sixty randomly selected census tracts formed the 2017 sample, which included 3200 households. Census tracts were previously established based on the division of each municipality into smaller regions with a steady population of 300 families. We then arbitrarily selected the cities, census tracts, and households that would participate. After a census tract was selected, the location of a cluster made of 20 houses in line was randomly defined, and the starting point of each cluster (the first household to be visited) was randomly selected utilizing ArcGIS, version 10.5, a software used for geoprocessing. In each household, information was obtained about all children living in that household from mostly the mother’s (97.2% were mother’s) or primary caregiver’s report. After the interview, the child’s anthropometric measurements were obtained by the trained staff. If a caregiver had multiple offspring in the household, all were included. All data were collected using paper forms and were double entered on Epi Info 2000 (CDC, Atlanta, GA, USA, 2011). Written informed consent was obtained from all participating primary caregivers for their and their child’s participation in the study. The PESMIC survey was approved by the Research Ethics Committee in Brazil.

### 2.2. Assessment

Standardized questionnaires were administered to the mother or the head of the household. Family income was evaluated through a direct interview with the caregiver, using the categories of the Brazilian scale of purchase power, which estimates family income by the number of assets [17]. Child nutritional status was assessed by standardizing the child’s weight and height by age and sex using the WHO reference curves [18].

Positive parenting behaviors were conceptualized in areas of interactive play, social development and speech and language interactions. The WHO and UNICEF guidelines were used as the model for the development of the evaluated parenting practices [19]. These guidelines identified twelve family and community practices considered vitally important to ensure survival, reduce morbidity, and promote healthy child growth and development through appropriate care, including talking, playing, and providing a stimulating environment. Considering that some parenting practices are essential for all ages (zero to six years old), while others are more important for specific age groups, we developed age-specific items in the questionnaire about parenting, as well as including general items that were asked to all age groups (Supplementary Box). Mothers/caregivers were asked to answer questions about what happened in the last three days of the children’s routine, while for parenting practices, which were asked of all age groups, mother/caregivers were asked to answer questions about what happened in the last seven days of the child’s routine, before the interview. For example, for general interactive play, all caregivers were asked, “In the last week, did you usually play with your child?”, and for the age-specific assessment of children aged 0–1 years, caregivers were asked if “In the last three days, did you play with your child using objects that make sounds?” and “In the last three days, did you play with your child using objects that roll?”. For age-specific speech and language interactions, for example, caregivers were asked, for children aged 0–1, “Did you sing songs or nursery rhymes to your child in the last three days?” and, for children aged 4 to 6, “Did you help teach your child to read in the last three days?”

We evaluated child development using the Ages and Stages Questionnaire Version 3 [20], which was validated in Brazil (ASQ-BR) [21,22]. Assessment was performed only in participants aged up to 66 months, as the ASQ has been developed only up for this age group. We measured five domains of child development: fine motor coordination, communication, broad motor coordination, problem solving, and the personal–social domains [20]. As suggested by the developers, a child’s domain score was excluded from analysis if more than 2 items of that specific domain assessment were skipped. If 1 or 2 items in one domain were skipped, an adjusted score was laid out by calculating the mean score for the completed items, and then replacing the skipped item with the calculated score [20].

The WHO definition of malnutrition was considered to establish the malnutrition variable. This definition includes stunting (low height for age), wasting (low weight for height), and underweight (low weight-for-age). We measured the weight to the nearest 0.1 kg with the use of a digital scale (SECA^®^, Hamburg, Germany). We obtained the length of children under 24 months of age to the nearest 0.1 cm with a length board, while the height of children > 24 months was measured with the use of a portable stadiometer to the nearest 0.1 cm.

All data were collected by interviewers were explicitly trained for 20 h by medical professionals who were experienced with anthropometric measures and ASQ-BR.

### 2.3. Statistical Analysis

Descriptive statistics adjusted for clustering by census tracts are presented. The total number of general parenting practices were classified into two categories: 3 or more positive parenting practices or fewer than 3 positive parenting practices, based on the distribution of the variable. Age- and sex-standardized scores of the ASQ-BR [23] for children aged ≥5 months were analyzed. For children aged <5 months, U.S. standards were used [24]. ASQ-BR age- and sex-standardized score were categorized as <−2 SD to indicate a positive screening for developmental delay. Chi-square tests that accounted for clustering by census tract and household were performed to test the association between general positive parenting practices and categorized child development scores. Multivariable generalized linear models that accounted for clustering by census tract and household and that used robust SE (standard errors) (to deal with non-normal distributions) were used to assess the association of general and age-specific parenting practices with ASQ-BR domain scores. A theoretical model was built, based on nurturing care models, which considered sociocultural factors (maternal education), degree of poverty (monthly income and assets possession), biological risk factors (malnutrition), toxic stress exposure (adverse childhood experiences), and parenting practices as the main determinants of child development [25,26]. Therefore, adjusted models that included covariates for the child’s age, sex, social class, malnutrition, maternal education, and interviewer are presented. We used pairwise deletion method to deal with missing data, and the posterior sensitivity analysis using missing values in determinants we carried out suggested minimal risk of bias. All study data were analyzed using SPSS, version 23.

### 2.4. Ethics

All participating mothers/caregivers issued written informed consent before the interviews. Written consent was also given by the mothers/caregivers on behalf of their children. The PESMIC survey was approved by the Research Ethics Committee in Brazil, with approval number CAAE 73516417.4.0000.5049.

## 3. Results

The baseline characteristics of the study participants, comprising 3566 caregiver/child pairs, are shown in Table 1. The mean maternal age was 28.6 years, and 22.3% were single and 68.2% were homemakers, with 4.4 years of schooling on average. The mean child age was 31.8 (SD = 23.1) months. The sample was equally distributed between the sexes, and 8.2% of the children were stunted.

**Figure 1:**
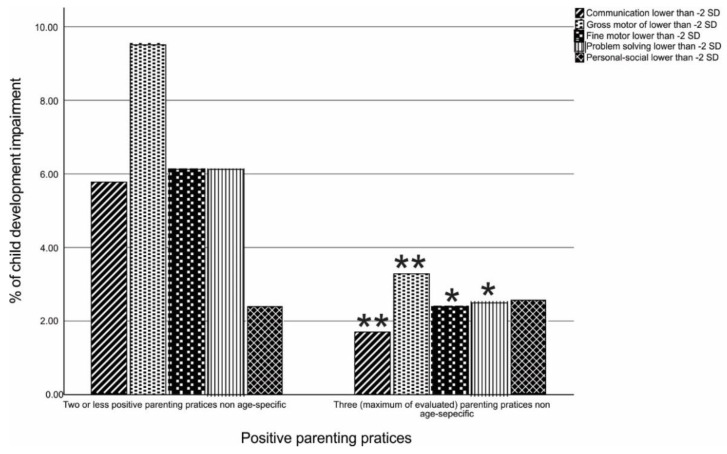
Percentage of children with developmental impairment in groups with all positive parenting behaviors present or not. * *p*-value = 0.001. ** *p*-value < 0.001.

Regarding parenting practices, 88.8% of the caregivers endorsed all three general positive parenting behaviors. In terms of ASQ developmental scores, 9.2% had at least one domain that showed a positive screening for developmental delay. Figure 1 shows the prevalence of positive screening for child development impairment per domain. For all domains, except for the personal–social, the prevalence of developmental delay was approximately three times higher in children whose caregivers did not report all three assessed general positive parenting behaviors (*p*-values < 0.05) (Figure 1).

### Association of Positive Parenting Behaviors and Child Development

The multivariate adjusted analyses that assessed the relationship of the number of age-specific PPBs with child development domain scores stratified by child age are summarized in Table 2. For infants aged 0 to 1 year, the number of age-specific PPBs was associated with all evaluated development domains; the communication (standardized mean difference for each additional PPB (SD) = 0.32, 95% CI (0.24–0.41)) and problem solving (SD = 0.38, 95% CI (0.24–0.52)) domains showed the highest magnitude of association. For children aged 1–2 years, all except the communication development domain score were associated with the number of PPBs (Table 2).

As for children aged 3–4 years, the age-specific PPBs were associated with all domains. Each additional specific PPB was associated with an increase of 0.12 SD for communication scores (95% CI: 0.05–0.18), 0.08 SD for gross motor (95% CI: 0.00–0.16), 0.16 SD for fine motor domain (95% CI: 0.10–0.23), 0.10 SD for problem-solving (95% CI: 0.03–0.17), and 0.12 SD for the personal–social domain (95% CI: 0.04–0.20). For children aged 4–6 years, age-specific PPBs were associated with the communication (SD = 0.22 (0.13–0.32)), problem solving (SD = 0.21 (0.10–0.32)), and personal–social domain scores (SD = 0.26 (0.17–0.36)).

The findings of the multivariable adjusted models that evaluated the association of specific PPBs with child development outcomes are shown in Supplementary Tables S1 and S2. For children aged up to one year, playing with small toys or toys that make noises was associated with better child development, with large effect sizes for all domains. For small toys, the standardized mean difference (SMD) was 0.94 (95% confidence interval (CI) 0.74–1.13); it was higher for the communication domain, 0.51 (CI 95% 0.3–0.73) higher for the gross motor domain, 0.46 (CI 95% 0.29–0.63) higher for the fine motor domain, 0.89 (CI 95% 0.57–1.21) higher for the problem-solving domain, and 0.31 (CI 95% 0.18–0.43) higher for the personal–social domain. For children aged 3 to 4 years, drawing/painting with the child was associated with a large positive association with the communication domain (0.43 (CI 95% 0.18–0.67, *p*-value 0.001)), fine motor domain (0.36 (0.1–0.62, *p*-value 0.007)) and personal–social domain scores (0.34 (CI 95% 0.05–0.62, *p* value 0.02)). Running with the child, singing to the child, taking the child for a walk, playing with rolling toys, or playing with little toys were not associated with improved child development for children aged 3 to 4 years. Finally, for children aged 4 to 6 years, all the evaluated specific positive parenting practices were associated with at least one child developmental domain.

## 4. Discussion

In this population-based cross-sectional study of 3566 children aged 0–6 years from the state of Ceará, Brazil, we identified that positive parenting behaviors were associated with improved child development across development domains. In addition, we found that the total number of age-specific PPBs was strongly associated with better child development outcomes.

The prevalence of all three evaluated general positive parenting practices was high in the studied population and was associated with an almost three-fold lower prevalence of impaired child development in all domains, except for the personal–social one. In addition, the magnitude of the associations found in this study (~0.3) is similar to that of the effect size found in intervention programs to strengthen parenting practices, as identified in a systematic review that evaluated 77 articles on interventions to improve the development of children aged 0 to 7 years [27]. Population assessment of parenting practices is rare in the context of Latin American countries; thus, the high prevalence of many parenting behaviors constitutes important epidemiological evidence to guide intervention programs. Families are central to Latino cultures, and this may explain the high level of positive parenting practices found in this population, as a study carried out with Latino families in the United States reported [28]. We theorize that children exposed to less frequent interactions with their caregivers compensate by developing a better relational capacity with other individuals, which may explain the absence of association of positive parenting practices with the personal–social domain.

We found that parenting behaviors involving toys appeared to be particularly associated with child development domains. It is important to emphasize that toys by themselves do not replace the parenting practice itself, i.e., it is the activity of playing with them that brings benefits to child development by facilitating the interaction between caregivers and their children, as recommended by the American Academy of Pediatrics guideline [29]. This is well exemplified by the importance of singing with the child, an interaction that does not require objects. For older children, playing with more complex toys was associated with better child development outcomes. For example, playing with puzzles was associated with better development in children aged 3–4 years. Although several studies have evaluated the beneficial effects of overall playing on child development, less attention has been paid to the types of toys used [30], and the evidence presented here is important to support the definition of priority types of toys for the adequate stimulation of child development in specific age strata, although more important than the type of toy is the interaction between parents and children. In addition, for the oldest evaluated children, the participation of caregivers in school learning activities, such as reading and writing, was associated with significant differences for higher development scores in all evaluated domains, except for gross motor development. Previous research has shown that this effect can be mediated by the children’s greater psychosocial maturity when exposed to appropriate parenting [31], which influences how the child will perform in high school. This is in agreement with the results found in our study for the problem-solving and personal–social domains. Moreover, caregivers who taught their children how to interact with other children and how to behave at school trained their children to have a better performance in the communication, problem-solving, and personal–social domains than children of caregivers who did not report doing so, with differences that reached almost one standard deviation.

## 5. Conclusions

Overall, positive parenting behaviors were independently associated with better child development outcomes in all studied domains in children from a Brazilian state with limited resources. Although the number of positive parenting behaviors was high in the studied population, associations were still identified. Additional research is needed to design interventions and programs aiming to improve or enhance parenting behaviors in the Brazilian context and evaluate their effect. The assessed behaviors originate in the family environment. Programs and interventions targeting families that encourage the specific parenting behaviors associated with the child development domains assessed in this study may universalize these practices and improve development outcomes.

### Limitations

This study has several limitations. First, although the ASQ-BR is a validated tool for child development impairment in Brazil, it is not a diagnostic instrument for child development impairment. Second, the study relied on the caregivers’ report of positive parenting behaviors, which may have led to some degree of overreporting, and we used a questionnaire for assessing parenting behaviors that, although based on UNICEF recommendations, was not yet validated. Third, although we used pairwise deletion to handle with missing data, which may have introduced bias; the posterior sensitivity analysis indicated low risk of bias. Fourth, the cross-sectional study design does not allow a direct determination of causal relationships due to the absence of an analysis of the child development trajectory. Furthermore, the results we found may not be generalizable to children in other settings, although the study was designed to be representative of all children of Ceará.

## Figures and Tables

**Table 1:** Characteristics of 3566 children aged 0–72 months of age that participated in the PESMIC survey in Ceará, Brazil.

Characteristics	Mean ± SD or *n* (%)
*Maternal and household characteristics*	
Maternal age, years	28.6 ± 7.2
Maternal schooling, years	4.4 ± 2.8
Marital status	
Single	780 (22.3)
Married	1159 (33.2)
Stable relationship	1370 (39.2)
Divorced	162 (4.6)
Widowed	22 (0.6)
Occupational status	
Homemaker	2365 (68.2)
Works outside the home	640 (18.4)
Works at home, for delivery services	351 (10.1)
Does not work	114 (3.3)
Monthly household income in Brazilian Reais in the past month	1090.4 ± 1017.9
Recipient of the Brazilian conditional cash transfer program	1943 (54.5)
*Child characteristics*	
Stunting (HAZ < −2)	293 (8.2)
Wasting (WHZ < −2)	76 (2.1)
Underweight (WAZ < −2)	107 (3.0)
Male child	1786 (50.0%)
Child age	31.8 ± 23.1
*Child ASQ-BR scores*	
Communication	52.2 ± 11.5
Gross motor	55.4 ± 9.3
Fine motor	49.7 ± 13.7
Problem solving	50.7 ± 12.5
Personal–social	50.1 ± 11.7

Notes: Values are means ± SDs or *n* (%); *n* = 3566. ASQ-3—Ages and Stages Questionnaire, version 3; SRQ-20—Self-Reported Questionnaire; HAZ—height-for-age Z score; WHZ—weight-for-height Z score; WAZ—weight-for-age Z score; PPB—Positive Parenting Behaviors; ASQ-BR—Ages and Stages Questionnaires Brazilian version.

**Table 2:** Association of the number of positive parenting behaviors with ASQ-3 child developmental domains stratified by child age.

		Communication		Gross Motor		Fine Motor		Problem Solving		Personal–Social	
		SMD for Each Additional Parenting Behavior ^1^	*p*-Value	SMD for Each Additional Parenting Behavior ^1^	*p*-Value	SMD for Each Additional Parenting Behavior ^1^	*p*-Value	SMD for Each Additional Parenting Behavior ^1^	*p*-Value	SMD for Each Additional Parenting Behavior ^1^	*p*-Value
*Children 0 to 1 year old*											
Number of age-specific positive parenting behaviors (4 maximum)											
Median	3	0.32 (0.24, 0.41)	<0.001	0.18 (0.11, 0.25)	<0.001	0.14 (0.06, 0.22)	<0.001	0.38 (0.24, 0.52)	0.001	0.11 (0.06, 0.17)	<0.001
(IQR)	(1–4)										
*Children 1–2 years old*											
Number of age-specific positive parenting behaviors (5 maximum)											
Median	5	0.05 (0.00, 0.10)	0.06	0.06 (0.02, 0.10)	<0.006	0.08 (0.02, 0.14)	<0.01	0.12 (0.06, 0.17)	<0.001	0.09 (0.03, 0.14)	<0.002
(IQR)	(4–5)										
*Children 3–4 years old*											
Number of age-specific positive parenting behaviors (6 maximum)											
Median	6	0.12 (0.05, 0.18)	<0.001	0.08 (0.00, 0.16)	<0.001	0.16 (0.10, 0.23)	<0.001	0.10 (0.03, 0.17)	<0.001	0.12 (0.04, 0.20)	<0.001
(IQR)	(4–6)										
*Children 4–6 years old*											
Number of age-specific positive parenting behaviors (5 maximum)											
Median	5	0.22 (0.13, 0.32)	<0.001	0.19 (−0.02, 0.39)	<0.07	0.13 (−0.3, 0.29)	<0.11	0.21 (0.10, 0.32)	<0.001	0.26 (0.17, 0.36)	<0.001
(IQR)	(4–5)										

^1^ Multivariable model includes adjustment for sex, income, malnutrition, toxic stress, maternal education, and interviewer.

## Data Availability

The datasets used and/or analyzed during the current study are available from the corresponding author on reasonable request.

## Supplementary Materials

The following supporting information can be downloaded at: https://www.mdpi.com/article/10.3390/children9081246/s1, Supplementary Box. Questionnaire used to appraise the positive parenting behaviors, Table S1: Association of individual positive parenting behaviors with ASQ-BR domain scores in children aged 0–2 years and Table S2: Association of individual positive parenting behaviors with ASQ-BR domain scores in children aged 3–6 years.
