# Supplementary material for: Positive Parenting Behaviors and Child Development in Ceará, Brazil: A Population-Based Study
Source: Children (Basel). 2022 Aug 18;9(8):1246. doi: 10.3390/children9081246 (PMC9406953; doi:10.3390/children9081246)
Supplement: Supplementary file 1 [file children-09-01246-s001.zip › children-1838081-supplementary.pdf]

Supplementary Material:

Supplementary box. Questionnaire used to appraise the positive parenting behaviors.

**For children of all age groups:**		
In the last week, did you or the child's father:		
PB79	Play with the child	1 - Yes 2 - No
PC79	Talk to the child	1 - Yes 2 - No
PP79	Walk with the child	1 - Yes 2 - No
**Only for children under 1 year of age:**		
For the last three days, did you, or another person in your family over 15 years of age, do any of these things with the child:		
BR80	Played with toys that make sounds, noise, play	1 - Yes 2 - No
	songs	1 - Yes 2 - No
CA80	Sang with the child	1 - Yes 2 - No
JB80	Threw a ball or rolling objects with the child	1 - Yes 2 - No
BP80	Played with Small Toys with the child	
**Only for children aged 1 to 2 years:**		
For the last three days, did you, or another person in your family over 15 years of age, do any of these things with the child:		
CA81	Sang with the child	1 - Yes 2 - No
JB81	Played with balls with the child	1 - Yes 2 - No
BP81	Played with small toys with the child	1 - Yes 2 - No
DP81	Drew/painted with the child	1 - Yes 2 - No
PA81	Took the child for a walk	1 - Yes 2 - No
**Only for children aged 3 to 4 years:**		
For the last three days, did you. or another person in your family over 15 years of age, do any of these things with the child:		
CO82	Ran with the child	1 - Yes 2 - No
CA82	Sang with the child	1 - Yes 2 - No
JB82	Played ball with the child	1 - Yes 2 - No
BP82	Played with small toys with the child	1 - Yes 2 - No
DP82	Drew/painted with the child	1 - Yes 2 - No
PA82	Took the child for a Walk	1 - Yes 2 - No
QB82	Played with puzzles with the child	1 - Yes 2 - No
**Only for children aged 4 to 5 years:**		
Did you or someone else in your family do any of these things with the child:		
EA83	Taught the alphabet (the letters)	1 - Yes 2 - No
EL83	Taught to read and/or write	1 - Yes 2 - No
ES83	Taught the child how to play with other children	1 - Yes 2 - No
EC83	Taught the child how to behave at school	1 - Yes 2 - No
AB83	Rode a bicycle, horse, etc. with the child	1 - Yes 2 - No

Supplementary Table S1. Association of individual positive parenting behaviors with ASQ-BR domain scores in children aged 0-2 years

Measure		n	Communication: Multivariable adjusted[a] standardized mean difference	Communication: *p*	Gross motor: Multivariable adjusted[a] standardized mean difference	Gross motor: *p*	Fine motor: Multivariable adjusted[a] standardized mean difference	Fine motor: *p*	Problem-solving: Multivariable adjusted[a] standardized mean difference	Problem-solving: *p*	Personal–social: Multivariable adjusted[a] standardized mean difference	Personal–social: *p*
Children aged up to 1 year												
Played with toys that make sounds												
	Yes	504	0.59 (0.39, 0.79)	**< 0.001**	0.30 (0.10, 0.50)	**0.003**	0.22 (0.05, 0.40)	**0.01**	0.63 (0.32, 0.94)	**< 0.001**	0.26 (0.13, 0.39)	**< 0.001**
	No	285	Ref		Ref		Ref		Ref		Ref	
Sang with the child												
	Yes	682	0.22 (-0.1, 0.54)	0.17	0.18 (-0.1, 0.47)	0.22	0.17 (-0.14, 0.49)	0.27	0.7 (0.11, 1.3)	**0.02**	0.05 (-0.13, 0.25)	0.55
	No	102	Ref		Ref		Ref		Ref		Ref	
Played with a ball or rolling toys												
	Yes	262	0.74 (0.58, 0.91)	**< 0.001**	0.4 (0.23, 0.56)	**< 0.001**	0.14 (-0.01, 0.31)	0.07	0.69 (0.40, 0.98)	**< 0.001**	0.37 (0.25, 0.48)	**< 0.001**
	No	523	Ref		Ref		Ref		Ref		Ref	
Played with other small toys												
	Yes	476	0.94 (0.74, 1.13)	**< 0.001**	0.51 (0.30, 0.73)	**< 0.001**	0.46 (0.29, 0.63)	**< 0.001**	0.89 (0.57, 1.21)	**< 0.001**	0.31 (0.18, 0.43)	**< 0.001**
	No	309	Ref		Ref		Ref		Ref		Ref	
Children aged 1-2 years												
Sang with the child												
	Yes	1165	0.17 (0.01, 0.34)	**0.03**	0.00 (-0.12, 0.13)	0.90	0.16 (0.00, 0.32)	0.06	0.28 (0.11, 0.45)	**0.001**	0.24 (0.06, 0.41)	**0.008**
	No	148	Ref		Ref		Ref		Ref		Ref	
Drew or painted with the child												
	Yes	869	0.00 (-0.09, 0.1)	0.88	0.1 (0.01, 0.19)	**0.02**	0.26 (0.14, 0.37)	**< 0.001**	0.13 (0.02, 0.23)	**0.01**	0.02 (-0.08, 0.13)	0.65
	No	439	Ref		Ref		Ref		Ref		Ref	

Played with a ball or rolling toys											
Yes	979	0.2 (0.09, 0.31)	**< 0.001**	0.16 (0.05, 0.26)	**0.003**	0.11 (-0.01, 0.24)	0.08	0.19 (0.08, 0.31)	**0.001**	0.22 (0.09, 0.35)	**0.001**
No	329	Ref		Ref		Ref		Ref		Ref	
Took for a walk											
Yes	1234	0.21 (-0.03, 0.45)	0.09	0.04 (-0.15, 0.24)	0.67	0.02 (-0.23, 0.28)	0.87	0.22 (0.00, 0.45)	0.06	0.41 (0.18, 0.64)	**< 0.001**
No	74	Ref		Ref		Ref		Ref		Ref	
Played with other small toys											
Yes	1214	0.29 (0.09, 0.50)	**0.004**	0.07 (-0.09, 0.24)	0.37	0.34 (0.12, 0.56)	**0.002**	0.26 (0.09, 0.43)	**0.002**	0.04 (-0.12, 0.21)	0.60
No	94	Ref		Ref		Ref		Ref		Ref	

Supplementary Table S2. Association of individual positive parenting behaviors with ASQ-BR domain scores in children aged 3-6 years

		Communication		Gross motor		Fine motor		Problem-solving		Personal–social	
**Measure**	**n**	**Multivariable adjusted[a]**	***p***	**Multivariable adjusted[a]**	***p***	**Multivariable adjusted[a]**	***p***	**Multivariable adjusted[a]**	***p***	**Multivariable adjusted[a]**	***p***
Children 3-4 years old											
Ran with the child											
Yes	431	0.14 (-0.02, 0.32)	0.09	0.22 (0.00, 0.44)	0.05	0.13 (-0.08, 0.35)	0.22	0.08 (-0.10, 0.27)	0.37	0.15 (-0.06, 0.37)	0.17
No	189										
Draw/painted											
Yes	505	0.43 (0.18, 0.67)	**0.001**	0.17 (-0.11, 0.45)	0.29	0.36 (0.10, 0.62)	**0.007**	0.19 (-0.04, 0.44)	0.11	0.34 (0.05, 0.62)	**0.02**
No	113										
Sang with the child											
Yes	514	0.15 (-0.06, 0.37)	0.17	0.17 (-0.10, 0.45)	0.23	0.27 (-0.01, 0.56)	0.07	0.09 (-0.13, 0.33)	0.40	0.11 (-0.14, 0.36)	0.38
No	104										
Took for a walk											
Yes	563	0.02 (-0.19, 0.25)	0.80	0.1 (-0.2, 0.4)	0.52	0.08 (-0.22, 0.39)	0.59	0.02 (-0.21, 0.25)	0.85	0.07 (-0.22, 0.37)	0.61
No	55										
Played with a ball or rolling toys											
Yes	413	0.1 (-0.05, 0.26)	0.19	0.04 (-0.13, 0.22)	0.64	0.14 (-0.05, 0.35)	0.16	0.03 (-0.13, 0.21)	0.67	0.16 (-0.03, 0.37)	0.10
No	205										
Played with puzzles											
Yes	236	0.16 (0.02, 0.29)	**0.02**	0.19 (0.03, 0.34)	**0.02**	0.38 (0.20, 0.57)	**< 0.001**	0.08 (-0.08, 0.24)	0.34	0.32 (0.14, 0.50)	**< 0.001**
No	382										
Played with small toys											
Yes	530	0.05 (-0.14, 0.25)	0.61	-0.09 (-0.32, 0.13)	0.42	0.21 (-0.06, 0.49)	0.13	0.04 (-0.19, 0.29)	0.67	0.06 (-0.20, 0.33)	0.64
No	88										
Children 4-6 years old											

Taught the alphabet										
Yes	787	0.45 (0.15, 0.75)	**0.003**	0.35 (-0.15, 0.85)	0.17	0.71 (0.13, 1.30)	**0.02**	0.64 (0.30, 0.97)	**< 0.001**	0.42 (0.15, 0.70)	**0.002**
No	76										
Taught to read and/or write										
Yes	769	0.36 (0.11, 0.60)	**0.004**	0.24 (-0.15, 0.65)	0.23	0.68 (0.13, 1.23)	**0.01**	0.51 (0.21, 0.81)	**0.001**	0.46 (0.21, 0.71)	**< 0.001**
No	94										
Taught how to interact with other children										
Yes	819	0.56 (0.17, 0.95)	**0.005**	0.53 (-0.27, 1.34)	0.20	0.18 (-0.54, 0.91)	0.61	0.68 (0.21, 1.15)	**0.005**	0.63 (0.19, 1.08)	**0.005**
No	43										
Taught how to behave at school										
Yes	827	0.54 (0.09, 0.99)	**0.02**	0.36 (-0.47, 1.21)	0.39	0.24 (-0.62, 1.11)	0.58	0.85 (0.33, 1.38)	**0.001**	0.58 (0.14, 1.01)	**0.01**
No	36										
Rode bikes or horses with the child										
Yes	513	0.46 (0.30, 0.63)	**< 0.001**	0.28 (-0.01, 0.57)	**0.07**	0.56 (0.19, 0.93)	**0.003**	0.37 (0.17, 0.56)	**< 0.001**	0.28 (0.1, 0.46)	**0.002**
No	350										
